# Triaxial Creep Mechanical Behaviors and Creep Damage Model of Dolomitic Limestone Material under Multi-Stage Incremental Loading

**DOI:** 10.3390/ma16051918

**Published:** 2023-02-25

**Authors:** Xingkai Wang, Wansheng Wei, Yong Niu, Caichu Xia, Leibo Song, Guansheng Han, Zheming Zhu

**Affiliations:** 1 School of Civil Engineering, Shaoxing University, Shaoxing 312000, China; 2 State Key Laboratory of Coal Mining and Clean Utilization, Beijing 100013, China; 3 Key Laboratory of Rock Mechanics and Geohazards of Zhejiang Province, Shaoxing University, Shaoxing 312000, China; 4 College of Architecture & Environment, Sichuan University, Chengdu 610065, China

**Keywords:** triaxial compression test, creep experiment, creep failure characteristic, creep threshold, accelerated creep, nonlinear creep damage model

## Abstract

Dolomitic limestone is the main surrounding rock material in Yangzong tunnel engineering; the instantaneous mechanical properties and creep behaviors of limestone are significant for stability evaluation during the stages of tunnel excavation and long-term maintenance. Herein, four conventional triaxial compression tests were carried out to explore its instantaneous mechanical behavior and failure characteristics; subsequently, the creep behaviors of limestone subjected to multi-stage incremental axial loading at the confinements of 9 MPa and 15 MPa were studied by employing an advanced rock mechanics testing system (i.e., MTS815.04). The results reveal the following. (1) comparing the curves of axial strain–, radial strain–, and volumetric strain–stress under different confining pressures shows that these curves present a similar trend, whereas the stress drops during the post-peak stage decelerate with the increase in confining pressure, suggesting that the rock transits from brittleness to ductility. The confining pressure also has a certain role in controlling the cracking deformation during the pre-peak stage. Besides, the proportions of compaction- and dilatancy-dominated phases in the volumetric strain–stress curves differ obviously. Moreover, the failure mode of the dolomitic limestone is a shear-dominated fracture but is also affected by the confining pressure. (2) When the loading stress reaches a creep threshold stress, the primary and steady-state creep stages occur successively, and a higher deviatoric stress corresponds to a greater creep strain. When the deviatoric stress surpasses an accelerated creep threshold stress, a tertiary creep appears and then is followed by creep failure. Furthermore, the two threshold stresses at 15 MPa confinement are greater than that at 9 MPa confinement, suggesting that the confining pressure has an obvious impact on the threshold values and a higher confining pressure corresponds to a greater threshold value. Additionally, the specimen’s creep failure mode is one of “abrupt” shear-dominated fracturing and is similar to that under a conventional triaxial compression test at high confining pressure. (3) A multi-element nonlinear creep damage model is developed by bonding a proposed visco-plastic model in series with the Hookean substance and Schiffman body, and can accurately describe the full-stage creep behaviors.

## 1. Introduction

The instantaneous and creep mechanical properties of rock materials, which are associated with the long-term stability of underground tunnels and roadways, are study areas of great concern in rock mechanics [1,2,3,4,5,6,7,8,9,10,11,12,13,14]. Many studies have revealed the uniaxial and triaxial creep behaviors of soft rocks such as coal rock [5,12], salt rock [15,16], sandstone [17], and red bed mudstone [18]. The creep deformation of soft rock is obvious [8]; however, the hard rock is rarely associated with a large creep deformation, but the hard rock engineering also shows noteworthy time-dependent deformation under long-term high-ground stresses [13,14]. Jiang et al. [19], Xu et al. [20], Yang et al. [21], Zhao et al. [13], and Hu et al. [22] have reported the creep characteristics of hard marble, greenschist, diabase, lherzolite rock, and sandstone, respectively.

In addition, numerous creep models have been applied to describe the creep behaviors of different rocks. These models mainly comprise linear and nonlinear types [6]. The classical models comprise the Maxwell substance, Kelvin body, Bingham model, and Burgers model, which are composed of linear elements and cannot describe the tertiary creep behavior [8,9,23]. Therefore, lots of new nonlinear rheological elements should be proposed to present the accelerated rheological deformation properties [21,22,24]; these can then be connected with the traditional creep models to develop nonlinear creep models that can describe the total creep stages.

However, owing to the limitation of the high-pressure experiment instruments and the strict conditions for creep tests, there is still little research on the triaxial compression creep tests associated with hard rocks, especially on dolomitic limestone rock. In this work, the chosen limestone rock specimens were extracted from the Yangzong Tunnel located in Yunnan Province, Southwest China, which is a very long tunnel with a buried depth of 200–400 m. During the excavation process and long-term operation stage, the instantaneous mechanical properties and time-dependent deformation features of surrounding rocks should be revealed to serve the safe construction design and long-term operation of this tunnel engineering.

## 2. Experimental Method

**Figure 1:**
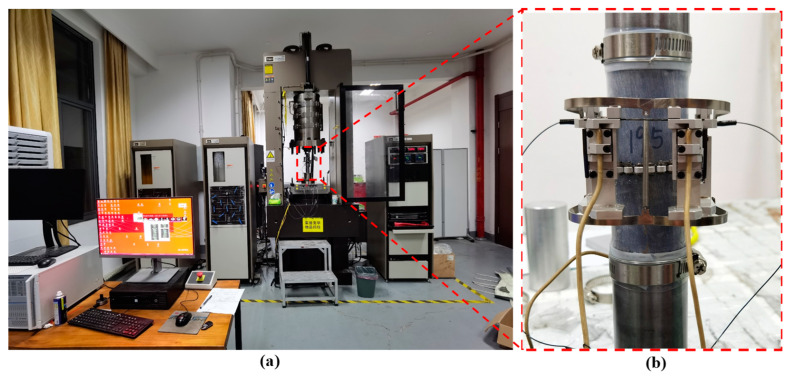
(**a**) Servo-controlled rock mechanics testing system (MTS 815.04) with high pressure (**b**) sealed specimen and radial deformation monitoring device.

Based on the method suggested by the International Society of Rock Mechanics and Rock Engineering (ISRM), the dolomitic limestone samples used in the experiments were cylinders of Φ50 mm × 100 mm. A servo-controlled rock mechanics testing system (MTS 815.04) with high rigidity and high pressure in Shaoxing University was used to carry out the conventional triaxial compression tests and creep experiments. The testing system is shown in Figure 1.

**Figure 2:**
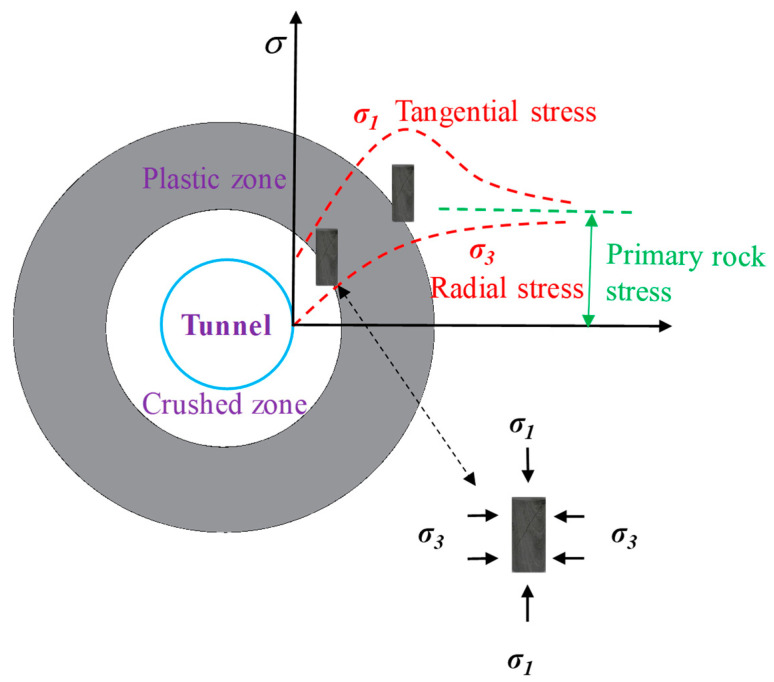
Redistribution of tunnel surrounding rock stress after excavation.

Four conventional triaxial compression tests were set first to probe the instantaneous mechanical characteristics of the rock specimens, which would provide a loading basis for the creep experiments. According to the in situ stress field and the stress redistribution (see Figure 2) of rock surrounding the tunnel which is excavated using drilling and blasting method, the radial stress of surrounding rock gradually increases from zero to the primary rock stress level as the distance increases; thus, the confining pressures were determined to be 0.5, 1, 9, and 15 MPa. Additionally, then, two creep tests with confining stresses of 9 and 15 MPa (i.e., the minimum and maximum horizontal in situ stresses) were designed to obtain the time-dependent mechanical properties of the rock material; the scheme is listed in Table 1.

**Figure 3:**
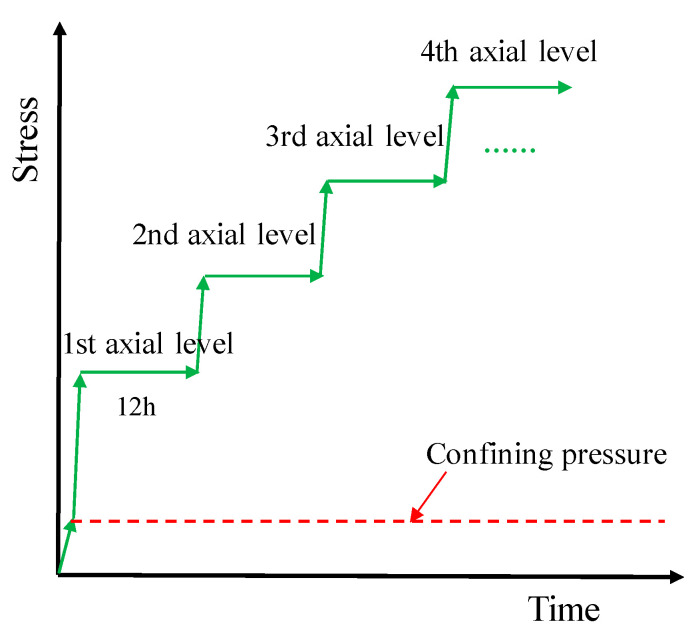
Loading method used in creep test.

During the conventional triaxial tests, a loading rate of 0.1 MPa/s for confining stress and an axial displacement rate of 0.001 mm/s were implemented. In the creep tests, a confining pressure loading rate of 0.1 MPa/s was applied to achieve the target value, which was then kept unchanged until the end of the experiment. Multi-level axial stresses were scheduled in the creep tests. For example, in Creep Test 1, the first axial stress was 37 MPa, and a loading rate of 0.5 kN/s was adopted to reach the target axial stress, and then remained constant for 12 h; subsequently, the next level of axial stress (47 MPa) was applied until the specimen was damaged. The loading process is shown in Figure 3. All the axial and confining pressures and axial and radial strains of the samples were monitored and recorded in real time, using the pressure transducers and axial and circumferential extensometers provided by the rock mechanics testing system.

## 3. Experimental Results Analysis and Discussion

### 3.1. Instantaneous Mechanical Characteristics

**Figure 4:**
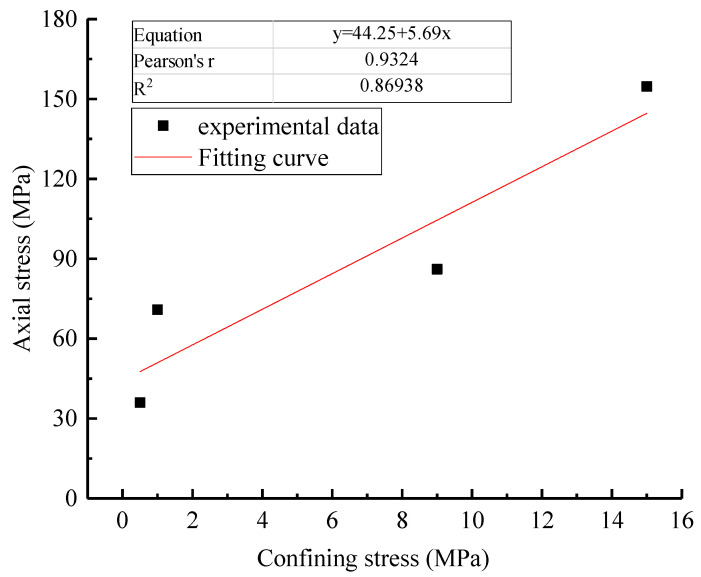
Relationship between confining pressure and peak axial pressure.

**Figure 5:**
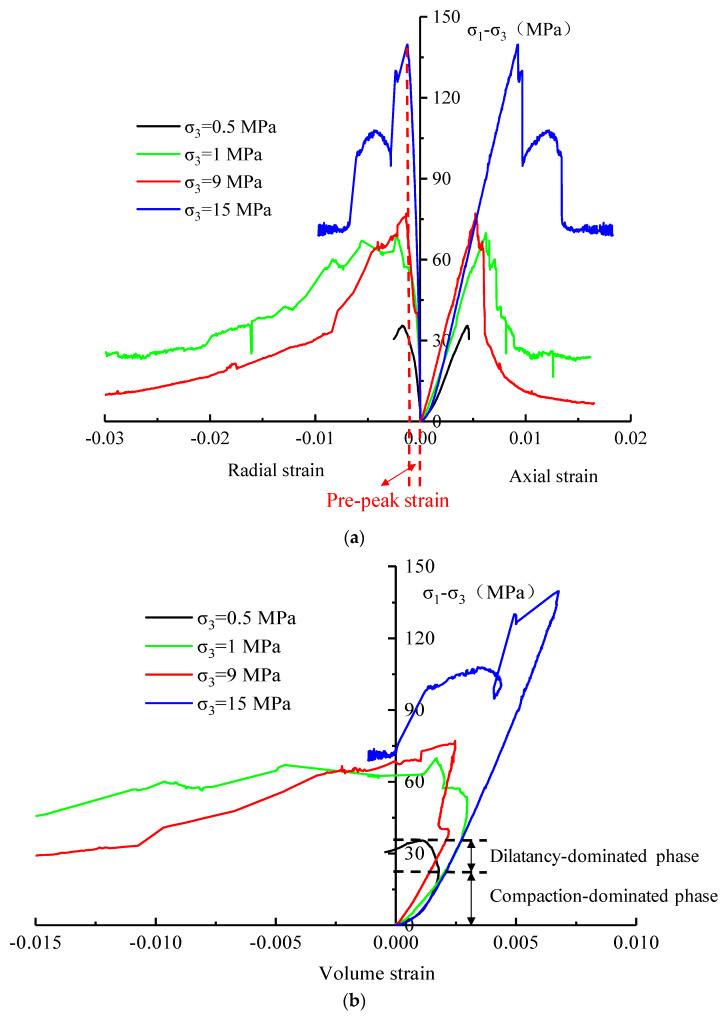
Axial strain-, radial strain-, and volumetric strain-deviatoric stress curves of the dolomitic limestone samples under different confining pressures.

The results of the conventional compression tests are shown in Figure 4 and Figure 5 and Table 2. The triaxial compressive strength of rock specimens increases linearly with the increase in confining pressure (see Figure 4), and then, according to Mohr–Coulomb triaxial strength theory, the internal friction angle and cohesion values could be obtained.

Figure 5 shows that the axial strain–deviatoric stress (calculated as *σ*_1_ − _3_) curves of the rock samples under different confining pressures present a similar trend, i.e., the rock mass undergoes at least four stages, consisting of the compaction closure of pores and micro fissures, linear elastic deformation, increase and expansion of microcracks, and post-peak failure. With the increase in confining pressure, the compaction stage was gradually shortened but did not disappear, which proves that the micro fissures of the limestone are relatively abundant. However, the stress drops during the post-peak stage decelerated with the increase in confining pressure, indicating that the rock transits from brittleness to ductility, and a higher confining pressure corresponds to a stronger plasticity and ductility; a similar phenomenon also appeared in other studies [25,26]. This phenomenon can be explained from the micro-scale: the influence of confining pressure on the mechanical properties of rock is reflected by an increase in the friction between crystals in the rock. As the confining pressure increases, more crystals are stressed to slip, more microcracks appear, and the crystal undergoes plastic deformation, leading to its transformation from brittleness to ductility on the macro-scale.

Besides, the radial strain–stress curves under different confining pressures are also similar. However, it can be seen by comparing the pre-peak strain that with the increase in confining pressure, the radial deformation decreased gradually, indicating that confining pressure has a certain role in controlling the cracking deformation of rock during the pre-peak stage.

In addition, Figure 5b reveals that the volumetric strain–stress curves differ obviously in shape during the pre-peak stages. Moreover, the evolution of volumetric strain is mainly determined by the continuous development and change of radial expansion and axial compression deformation of the sample. Under low confining pressure, the volume compaction-dominated phase occurred first and then was followed by the dilatancy-dominated phase during the pre-peak stage (see the volume strain curve at confining pressure of 0.5 MPa). However, under high confining pressure, the whole pre-peak stage mainly displayed volume compaction-dominated deformation. However, during the post-peak stage, the volume of rock samples under different confining pressures expanded continuously with the development of rock fractures.

**Figure 6:**
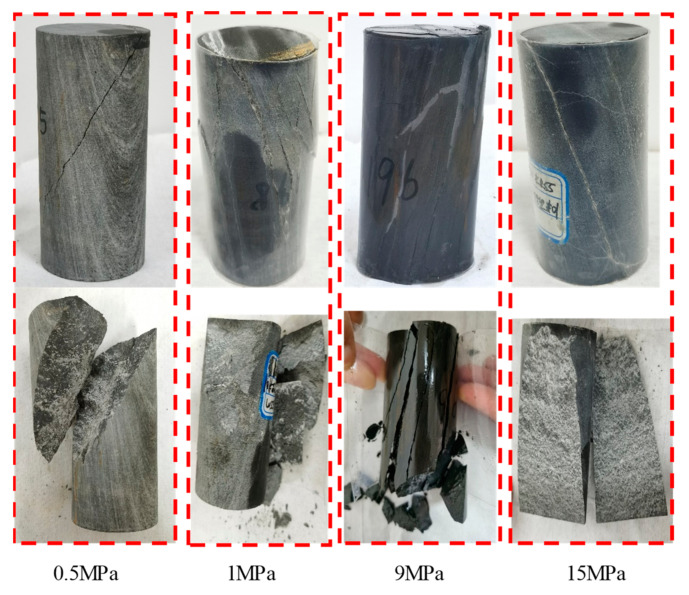
Failure modes of the dolomitic limestone samples under conventional compression tests at different confining pressures.

Figure 6 illustrates the failure characteristics of the dolomitic limestone samples under different confining pressures. When the confining pressure was 0.5 MPa, a single shear plane formed with an angle of about 45° to the axial direction of the sample; when the confining pressure was 1.0 MPa, the angle between the shear plane and the axial direction of the specimen was approximately 45°, but the specimen was broken by multiple shear planes. As the confining pressure rose to 9.0 MPa, the angle between the shear plane and the axial direction of the specimen was approximately 30°, and multi shear plane fracture occurred; when the confining pressure rose to 15.0 MPa, the angle between the shear plane and the axial direction of the specimen was approximately 30°, but the main shear fracture plane and other shear planes (part of the original joint planes split) occurred jointly, and the failure degree was intensified. The basic laws can be summarized as follows: under the conventional triaxial compression test, the failure mode of the dolomitic limestone is shear-dominated fracture. However, the failure characteristics are obviously affected by the confining pressure, i.e., with the increase in confining pressure, the included angle between the fracture surface and the axial direction of the sample decreases (which is different from the research results of sandstone [27]), and the fracture and original joint surface are developed and broken more fully.

### 3.2. Creep Mechanical Properties

**Figure 7:**
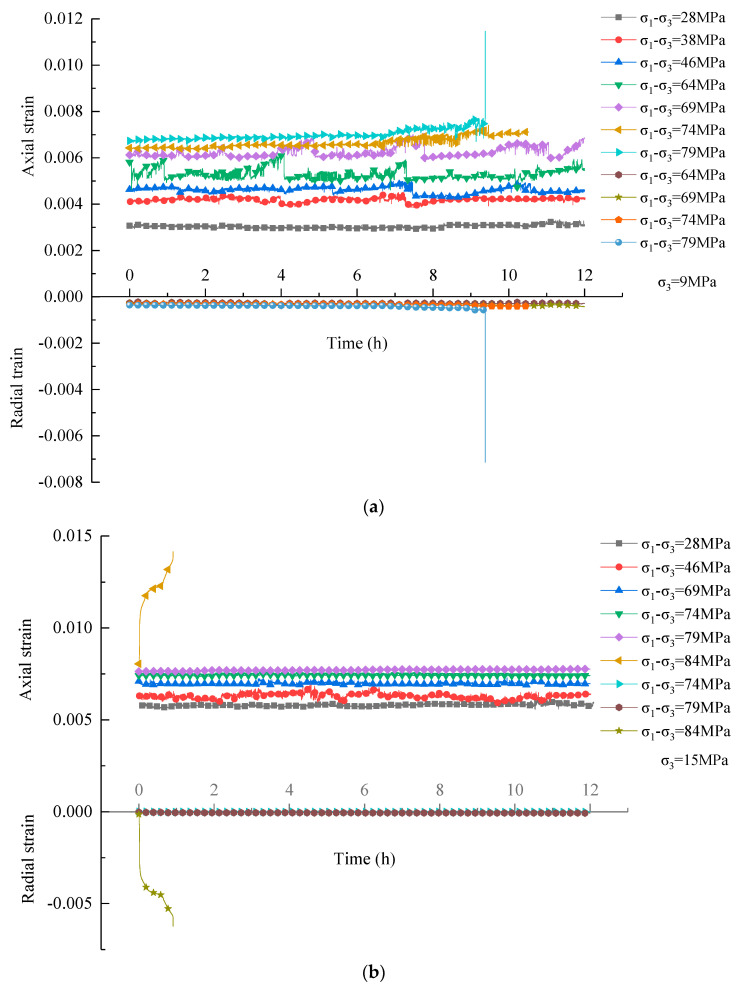
Creep curves of strain–time at confining pressures of (**a**) 9 MPa and (**b**) 15 MPa.

Figure 7 presents the creep behavior’s evolution with time under different stress levels at confining pressures of 9 MPa and 15 MPa. This paper only analyzes the evolution of axial strain because the evolution laws of radial and axial strains are similar. It can be seen from Figure 7a that under each level of deviatoric stress, instantaneous strain occurred first, but the creep strain did not increase with time before the deviatoric stress level of 69 MPa. When the deviatoric stress reached 69 MPa, the creep strain increased with time; primary creep and steady-state creep occurred successively, and a higher deviatoric stress corresponds to a greater creep strain. Under the last deviatoric stress level of 79 MPa, an accelerated creep stage occurred and was then followed by rock failure. The above results indicate that there is a creep threshold for the rock mass, which is in the range of 64 MPa to 69 MPa under a confining pressure of 9 MPa. When the applied deviatoric stress is less than this threshold, there is no creep deformation.

Similarly, according to Figure 7b, the creep strain did not increase apparently with time until the stress level reached 74 MPa, as the stress level continued to increase and the strain increased more significantly with time. Under the last level of deviatoric stress of 84 MPa, a significant tertiary creep appeared, the creep strain increased nonlinearly with time during this stage, and the rock ruptured when the strain reached a critical value. Thus, at the confining pressure of 15 MPa, the values of creep and accelerated creep threshold deviatoric stresses are 69–74 MPa and 79–84 MPa, respectively. The creep behaviors mentioned above are similar to those of hard and brittle rock [13,21,22]. It is worth noting that the creep threshold stress and accelerated creep threshold stress under a confining pressure of 15 MPa are greater than those under a confining pressure of 9 MPa, which suggests that the confining pressure has an obvious impact on the threshold values, and a higher confining pressure corresponds to a greater threshold value. High confining pressure is more conducive to reducing the possibility of creep and accelerated creep behaviors.

**Figure 8:**
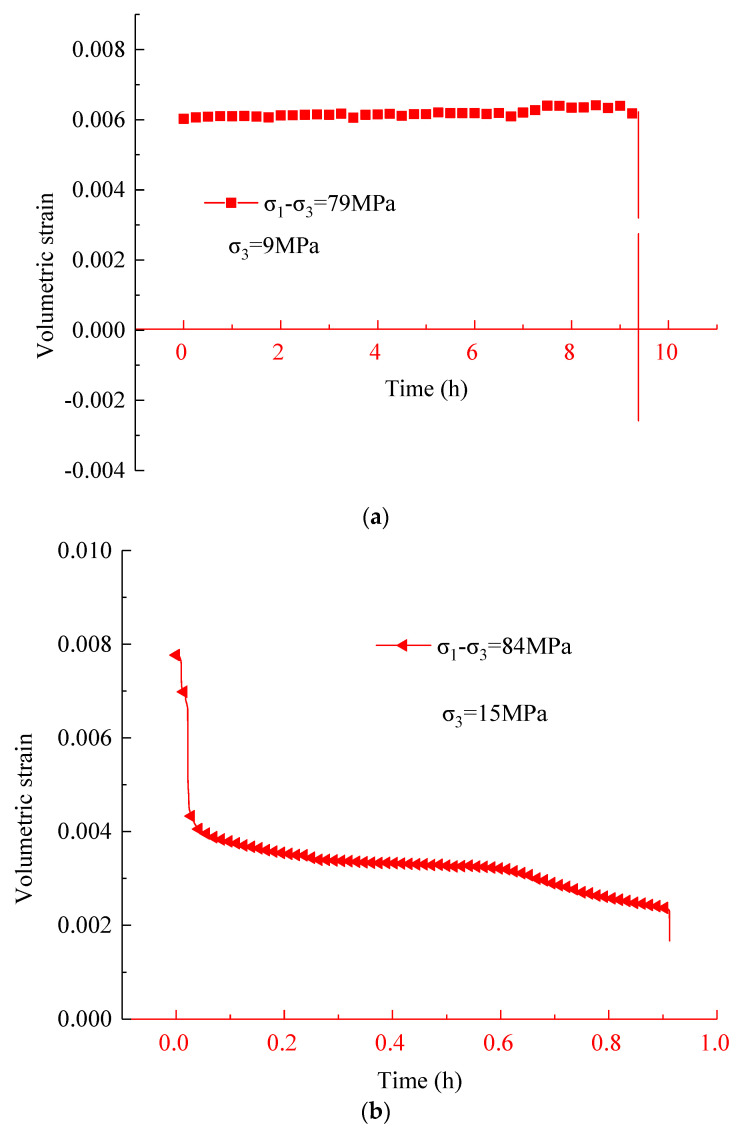
Creep curves of volumetric strain–time under the last deviatoric stress at confining pressures of (**a**) 9 MPa and (**b**) 15 MPa.

Besides, the evolution of volumetric strain versus time at the maximum stress level in the two tests is drawn in Figure 8, because the volumetric strain under other stress levels was generally dominated by compression. Figure 8 shows that during the triaxial compression creep test, the evolution trend of the creep curve of volumetric strain versus time of the sample was basically consistent with that of the curve of radial strain versus time, and had a typical three-stage feature. First, the compression volume strain decreased rapidly, then the compression volume strain decreased slowly. Finally the compression volume strain decreased rapidly and even the expansion volume strain occurred. The variation in volumetric strain is mainly due to the continuous development of damage and cracks in rock samples under high stress resulting in the continuous increase in radial strain; particularly, when the samples were close to complete failure, the lateral expansion deformation was the main factor.

**Figure 9:**
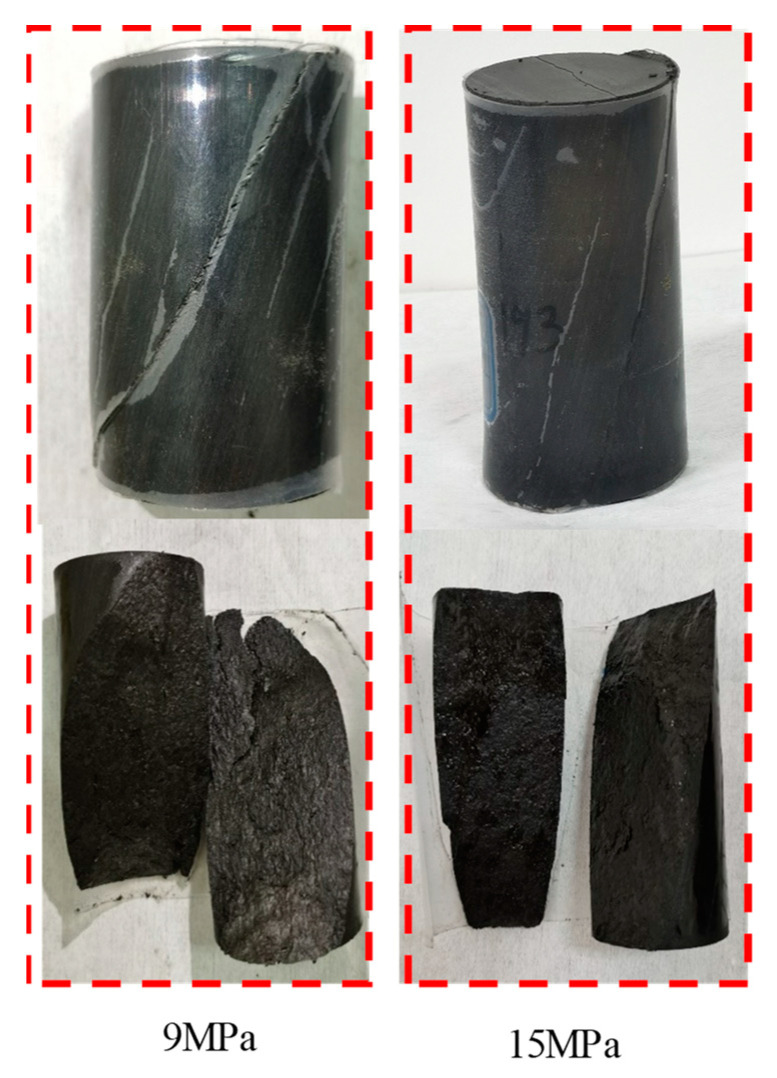
Failure characteristics of dolomitic limestone samples under triaxial creep compression tests with multi-stage loading at confining pressures of 9 MPa and 15 MPa.

Furthermore, Figure 9 illustrates the failure characteristics of dolomitic limestone samples under triaxial creep compression tests with multi-stage loading. The failure mode was a shear-dominated fracture with a fracture surface running through the whole specimen; the angle between the shear plane and the axial direction of the specimen was approximately 30°, which signifies an “abrupt” fracturing instability mode and is similar to the failure characteristics of the specimen under a conventional triaxial compression test at a high confining pressure. In addition, compared with the “progressive” creep failure modes of coal, salt, and other soft rocks [5,15,28], no obvious lateral expansion deformation and squeezing deformation occurred during the creep failure stage of dolomitic limestone.

### 3.3. Development of Nonlinear Creep Damage Model

**Figure 10:**
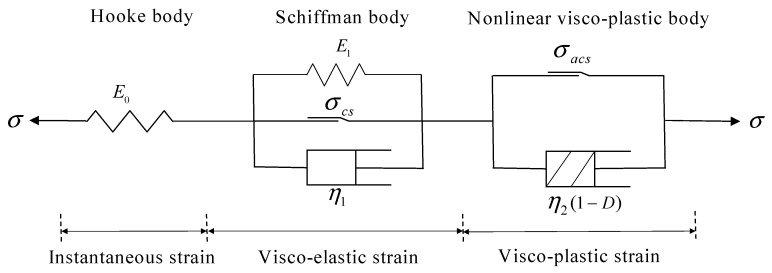
A new multi-element nonlinear creep damage model for dolomitic limestone.

Figure 10 shows a newly developed multi-element creep model adopted to describe the creep behaviors of the dolomitic limestone subjected to multi-stage incremental axial loading. According to the creep behaviors shown in Figure 7, a Hookean body is needed to express the instantaneous deformation of the rock specimen produced under each stress level. Besides, a Schiffman body, which is different from the Kelvin model (as there is a creep threshold stress), is used to exhibit the creep behavior when the stress level exceeds a certain value (i.e., *σ_cs_*). Furthermore, because the combined model cannot describe the nonlinear creep property [6,17,21,23], a nonlinear creep body should be established to describe the visco-plastic deformation that occurrs during the accelerated creep stage after the stress reaches a high threshold value (i.e., *σ_acs_*).

From the view of damage mechanics [6,9,23,24,29], rock creep failure is caused by the continuous damage and mechanical property deterioration of rock materials under long-term stress loading, and is accompanied by crack initiation, propagation, penetration, and slip. During the accelerated creep stage, the damage degree of rock will develop rapidly under high deviatoric stress, and the viscous coefficient will continue to decrease. Finally, the unrecoverable visco-plastic deformation increases nonlinearly, and the sample will break and fail. Herein, according to the Kachanov rheological damage theory [30], a nonlinear viscous damage element was proposed by introducing a damage variable considering time effect, i.e., the rheological damage variable of rock increases in a negative exponential function with time, and then the creep differential constitutive relation of the nonlinear viscous damage element can be derived as

(1) $${\dot{\varepsilon }}_{vd}=\frac{\sigma }{\eta_{2}(1-D)}=\frac{\sigma }{\eta_{2}[1-(1-e^{-\alpha t})]}$$

where ${\dot{\varepsilon }}_{vd}$ is the creep rate of nonlinear viscous damage element, $\eta_{2}$ is the initial viscous coefficient of the viscous element, *t* is creep time, and α indicates the rock damage index.

Then, the creep constitutive equation of the nonlinear viscous damage element can be obtained by integrating Equation (1), i.e.,

(2) $$\varepsilon_{vd}=\frac{\alpha \sigma }{\eta_{2}}\exp(\alpha t)+C$$

where *C* is the integral constant, when *t* is 0, $\varepsilon_{vp}$ is 0, and the value of *C* can be thus obtained as

(3) $$C=-\frac{\alpha \sigma }{\eta_{2}}$$

Additionally, then Equation (4) can be obtained by substituting Equation (3) into Equation (2), i.e.,

(4) $$\varepsilon_{vd}=\frac{\alpha \sigma }{\eta_{2}}\exp(\alpha t)-\frac{\alpha \sigma }{\eta_{2}}$$

Moreover, the three components in the new creep model should meet the following formulation:

(5) $$\left\{ \begin{array}{l} \sigma =\sigma_{part1}=\sigma_{part2}=\sigma_{part3}\\ \varepsilon =\varepsilon_{part1}+\varepsilon_{part2}+\varepsilon_{part3}\\ \sigma_{part1}=E_{0}\cdot \varepsilon_{part1}\\ \sigma_{part2}-\sigma_{cs}=E_{0}\cdot \varepsilon_{part2}+\eta_{1}\cdot {\dot{\varepsilon }}_{part2}\\ \sigma_{part3}-\sigma_{acs}=\eta_{2}\cdot {\dot{\varepsilon }}_{part2}\cdot e^{-\alpha t} \end{array}\right.$$

where *σ* and *ε* are the total deviatoric stress and the total strain of the creep model, respectively; *σ_part_*_1_, *σ_part_*_2_, and *σ_part_*_3_ are loading stresses for the first, second, and third parts in the model, respectively; *ε_part_*_1_, *ε_part_*_2_, and *ε_part_*_3_ are the strains for Parts 1, 2, and 3, respectively; *σ_cs_* indicates the creep threshold stress; *σ_acs_* indicates the accelerate creep threshold stress; *E* and *η* present the elastic and viscous parameters of the basic elements; and $\dot{\varepsilon }$ is the first derivative of strain and indicates deformation rate.

Finally, according to the constitutive equations of the Hookean substance, the Kelvin body, the plastic element and the Laplace transform and its inverse transform, the creep equation of the new nonlinear creep model could be derived as

(6) $$\varepsilon (t)=\left\{ \begin{array}{l} \frac{\sigma_{1}-\sigma_{3}}{E_{0}},\sigma <\sigma_{cs}\\ \frac{\sigma_{1}-\sigma_{3}}{E_{0}}+\frac{\sigma_{1}-\sigma_{3}-\sigma_{cs}}{E_{1}}\left[1-\exp(-\frac{E_{1}}{\eta_{1}}t)\right],\sigma_{cs}\leq \sigma <\sigma_{acs}\\ \frac{\sigma_{1}-\sigma_{3}}{E_{0}}+\frac{\sigma_{1}-\sigma_{3}-\sigma_{cs}}{E_{1}}\left[1-\exp(-\frac{E_{1}}{\eta_{1}}t)\right]+\\ \frac{\alpha (\sigma_{1}-\sigma_{3}-\sigma_{acs})}{\eta_{2}}\exp(\alpha t)-\frac{\alpha (\sigma_{1}-\sigma_{3}-\sigma_{acs})}{\eta_{2}},\sigma_{acs}\leq \sigma \end{array}\right.$$

It should be pointed out that when there are enough test samples, the empirical expressions of the two creep threshold stresses (i.e., *σ_cs_* and *σ_acs_*) concerning the confining pressure can be obtained, and can be used to estimate the critical stresses of creep occurrence and accelerated creep occurrence for limestone under different test conditions.

### 3.4. Parameters Identification of Creep Model

In order to verify the correctness of the new creep model and identify its parameters, it is necessary to perform parameter identification based on the experimental data. First, the instantaneous parameter *E*_0_ can be determined using Equation (7) and the elastic strain data under each stress level.

(7) $$E_{0}=\frac{\sigma_{1}-\sigma_{3}}{\varepsilon_{part1}}$$

**Figure 11:**
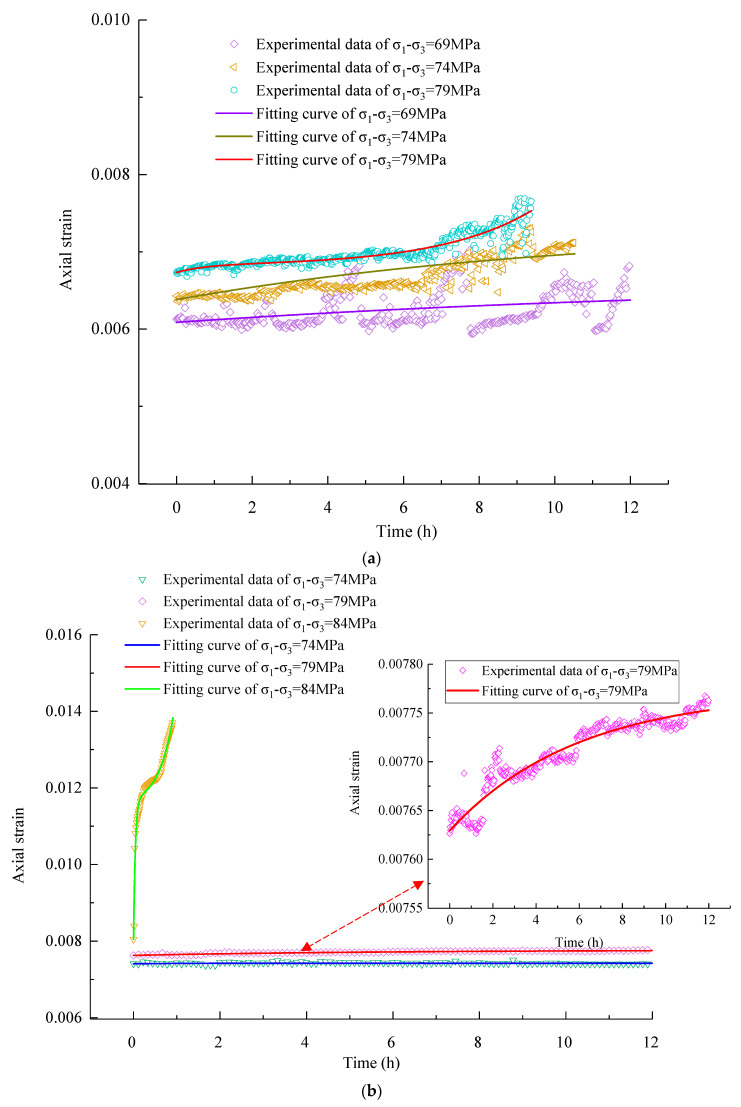
Comparison between the calculation curves and the experimental data under different axial stresses at confining stress of (**a**) 9 MPa and (**b**) 15 MPa.

Second, the parameters *E*_0_, *η*_1_, and *η*_2_ could be identified by fitting the creep curves using Equation (6) based on the Levenberg–Marquardt optimization algorithm in Origin software 2017. It should be noted that only the range value of the threshold stresses can be determined in this work; thus, in parameter identification, the lower stress limits of the range values were taken as a reference. Table 3 shows the identification results of all model parameters under different stress levels at two confining pressures. Moreover, Figure 11 shows that the development trend of prediction curves is highly consistent with that of creep test curves, which verifies that the established model is correct. In particular, the correlation index between the experimental result curves with total three creep stages and the theoretical curves of the proposed model in the two tests is 0.87 and 0.97, respectively.

**Figure 12:**
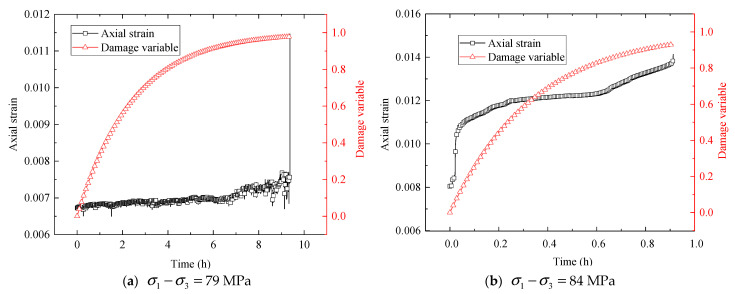
Evolution curves of damage variable– and strain–time under the last level of deviatoric stress at confining pressures of (**a**) 9 MPa and (**b**) 15 MPa.

Besides, the evolution curves of the damage variable and axial strain with time under the last deviatoric stress levels are presented in Figure 12. It can be seen that with the increase in creep time, the damage variable develops rapidly, and the visco-plastic strain increases nonlinearly. The final damage variable approaches 1, indicating that the rock is close to damage failure, and finally, creep failure occurs.

### 3.5. Parameters Analysis of Creep Model

**Figure 13:**
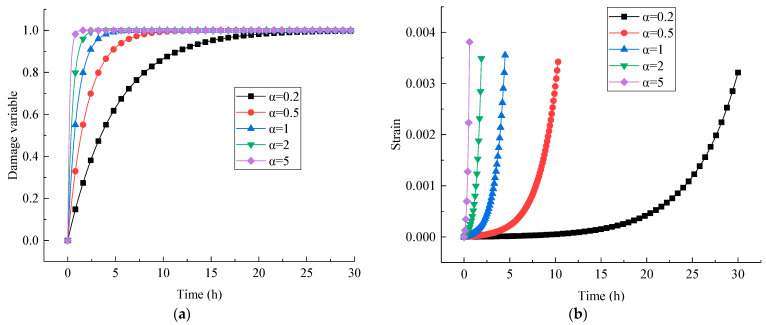
Effect of rock damage index *α* on the (**a**) damage variable and (**b**) strain curves under the last level of deviatoric stress with a confining pressure of 9 MPa.

To expand the universality of the model and facilitate its use for other projects, the influence rule of rock damage index *α* on the damage variable and accelerated creep curve is analyzed, as shown in Figure 13. The creep equation of the tertiary creep stage is arranged as Equation (8). Figure 13 reveals that the rock damage and the tertiary creep accelerate prominently, especially when the value of rock damage index *α* is between 0.2–1 and the influence is more significant. Since other creep parameters in Table 3 are commonly used in the traditional linear Kelvin model, their sensitivity analysis will not be repeated here.

(8) $$\varepsilon_{tertiary}=\frac{\alpha (\sigma_{1}-\sigma_{3}-\sigma_{acs})}{\eta_{2}}\exp(\alpha t)-\frac{\alpha (\sigma_{1}-\sigma_{3}-\sigma_{acs})}{\eta_{2}}$$

Furthermore, compared with the previous multi-element creep models [5,29], the creep damage model in this work does not need to segment the accelerated creep and initial two creep stages during parameter identification, which is also an advantage of the damage creep model. The physical meaning of the damage model is more clear and more concise. Therefore, the damage creep model proposed in this paper can be widely used to predict the creep behavior of brittle and hard rocks; however, the prediction of the creep threshold stress of the model needs more laboratory tests to be successful in future studies.

## 4. Conclusions

(1) The instantaneous mechanical behaviors and failure characteristics of dolomitic limestone were revealed using conventional triaxial compression tests. The results show that the axial strain–stress curves of the rock samples under different confining pressures presented a similar trend, whereas the stress drops during the post-peak stage decelerated with an increase in confining pressure, indicating that the rock transitions from brittleness to ductility, and a higher confining pressure corresponds to a stronger plasticity and ductility. Besides, comparison of the radial strain–stress curves shows that the confining pressure has a certain role in controlling the cracking deformation of rock during the pre-peak stage. Additionally, the volumetric strain–stress curves differ obviously in shape during the pre-peak stages, i.e., under low confining pressure, the volume compaction-dominated phase occurs first and is then followed by the dilatancy-dominated phase. However, under high confining pressure, the whole pre-peak stage mainly displayed volume compaction-dominated deformation. Moreover, the failure mode of the dolomitic limestone is a shear-dominated fracture but is also affected by the confining pressure; the included angle between the fracture surface and the axial direction of the sample decreases with the increase in confining pressure.
(2) The triaxial compression creep behaviors of limestone subjected to incremental loading at confining pressures of 9 MPa and 15 MPa indicate that the strain does not increase with time before a certain creep threshold stress. When the loading stress exceeds it, the primary creep and steady-state creep occurred successively, and a higher deviatoric stress corresponds to a greater creep strain. When the deviatoric stress outstripped an accelerated creep threshold stress, a significant tertiary creep appeared and then was followed by rock creep failure. Besides, the creep threshold stress and accelerated creep threshold stress under a confining pressure of 15 MPa are greater than those under confining pressure of 9 MPa, suggesting that a high confining pressure is more conducive to reducing the possibility of creep and accelerated creep behaviors. Moreover, the creep failure mode of dolomitic limestone samples is a shear-dominated fracture with a fracture surface running through the whole specimen, and the angle between the shear plane and the axial direction of the specimen is approximately 30°, which represents “abrupt” fracturing instability. This is similar to the failure characteristics revealed by a conventional triaxial compression test at a high confining pressure.
(3) Based on the creep characteristics of the dolomitic limestone specimens, a nonlinear visco-plastic body was proposed to reflect the tertiary creep deformation by introducing a viscous damage element considering the time effect; then, a new multi-element nonlinear creep damage model was developed by bonding the new visco-plastic body in series with the Hookean substance and Schiffman model, which could accurately describe the full-stage creep behavior. Besides, the influence rule of the rock damage index on the damage variable and accelerated creep curve was analyzed, and the advantage of the damage creep model was discussed.

These research results can provide an experimental basis and theoretical guidance for long-term stability analysis and maintenance of underground rock engineering.

## Figures and Tables

**Table 1:** Creep test scheme.

No.	Confining Pressure (MPa)	Axial Stress Levels (MPa)						
		1st	2nd	3rd	4th	5th	6th	7th
1	9	37	47	55	73	78	83	88
2	15	43	61	84	89	94	99	-

**Table 2:** Results of conventional triaxial compression tests.

Confining Pressure (MPa)	Compressive Strength (MPa)	Cohesion (MPa)	Internal Friction Angle (°)	Poisson Ratio
0.5	35.98	12.58	44.52	0.18
1.0	70.87			
9.0	86.06			
15.0	154.75			

**Table 3:** Parameter identification results of the testing specimen.

*σ*_3_ (MPa)	*σ*_1_ − *σ*_3_ (MPa)	*E*_0_ (GPa)	*σ_cs_* (MPa)	*E*_1_ (GPa)	*η*_1_ (GPa·h)	*σ_acs_* (MPa)	*η*_2_ (GPa·h)	*α*
9	69	11.33	64–69	9.18	146.39	74–79	-	-
	74	11.60		10.49	113.53		-	-
	79	11.74		149.16	111.24		125.20	0.41
15	74	9.99	69–74	370.72	388.67	79–84	-	-
	79	10.35		70.30	416.35		-	-
	84	10.43		4.09	0.17		93.27	2.93

## Data Availability

The data presented in this study are available on request from the corresponding author.
